# Remedy for Rheumatism

**Published:** 1884-07

**Authors:** 


### REMEDY FOR RHEUMATISM.

TO a medical man the announcement of a remedy for rheumatism is about as definite as a remedy for *illness*.. There are various forms of rheumatism, and any part of the body may be affected. It is therefore absurd to claim that the same remedy will relieve all cases. But there is a form of the complaint that is very common in malarial districts, for which we would suggest a treatment that has been highly commended by persons who have employed it.

Take one or two teaspoonfuls of *Rochelle salts* in half a glass of cold water; follow this immediately with a glass of lemonade, which contains all the juice of a large lemon, and the requisite quantity of sugar to render it pleasant to drink. This simple treatment may be employed once or twice a day, according to the severity of the rheumatism. We are assured that this plan will cure in eight to twelve days any rheumatism of miasmatic character. The treatment is indeed based on scientific principles. Citric acid separates any excess of bile from the blood, and the Rochelle salts hastens its elimination from the system. This form of rheumatism depends upon an unhealthy condition of the blood caused by malarial poison; consequently no form of treatment could be more rational than the one above suggested. And nothing can be more absurd than to expect to cure rheumatism with liniments and *massage* alone. These are simply useful in affording relief until more thorough remedies have time to act.

